# The Impact of COVID-19 on Levels of Adherence to the Completion of Nursing Records for Inpatients in Isolation

**DOI:** 10.3390/ijerph182111262

**Published:** 2021-10-27

**Authors:** Mercedes Fernández-Castro, José-María Jiménez, Belén Martín-Gil, María-Fé Muñoz-Moreno, María-José Castro, María-José Cao, María López

**Affiliations:** 1 Hospital Clínico Universitario de Valladolid, 47002 Valladolid, Spain; mefernandezc@saludcastillayleon.es (M.F.-C.); bmartingi@saludcastillayleon.es (B.M.-G.); mfmunozm@saludcastillayleon.es (M.-F.M.-M.); 2 Nursing Faculty, University of Valladolid, 47005 Valladolid, Spain; mariajose.castro@uva.es (M.-J.C.); mjcao@enf.uva.es (M.-J.C.); maria.lopez.vallecillo@uva.es (M.L.)

**Keywords:** nursing records, patient isolation, COVID-19, nursing care, nurses

## Abstract

The COVID-19 pandemic has led to an increased workload for nurses and organisational and structural changes, which have been necessary to meet the needs of inpatients in isolation. Aim: To describe the impact of the COVID-19 pandemic on levels of adherence to the completion of nursing records that document the risk of developing pressure ulcers, falling, and social vulnerability among hospitalised patients in isolation. Methods: Observational pre-post comparison study. Comparison between nursing records (the Braden, Downton, and Gijón scales) belonging to 1205 inpatients took place in two phases. Phase 1: 568 patients admitted in February 2020, prior to the COVID-19 pandemic, vs. phase 2: 637 patients hospitalised with COVID-19 in March–April 2020, during the peak of the first wave of the pandemic. This research adheres to the STROBE guidelines for the reporting of observational studies. Results: The degree of completion of the Braden, Downton, and Gijón scales decreased significantly in phase 2 vs. phase 1 (*p* < 0.001). The mean Downton and Gijón scale scores for patients admitted in phase 1 were higher compared to those of patients admitted in phase 2 (*p* < 0.001). The mean Braden scale score in phase 2 was higher than in phase 1 (*p* < 0.05). Conclusion: During the COVID-19 pandemic, there was a decrease in the completion of nursing records in the clinical records of patients in isolation. The levels of risk of developing PUs, falling, and social vulnerability of patients admitted to hospital were lower during the first wave of the pandemic.

## 1. Introduction

On 12 March 2020, the World Health Organisation declared the spread of the severe acute respiratory syndrome coronavirus 2 (SARS-CoV-2) to be a pandemic [1]. The number of confirmed cases worldwide has exceeded 49.7 million, while the number of deaths has risen to more than 1.2 million. In March 2020, during the first wave of the pandemic, the number of confirmed cases of COVID-19 (coronavirus disease 2019) in Spain was 78,797 compared to 693,282 worldwide. The number of new cases and deaths reported in Europe increased exponentially in November 2020. Spain emerged as the country with the sixth highest number of COVID-19 cases worldwide, with more than 2000 new cases per million inhabitants [1]. 

In this context, hospital managers were required to make considerable organisational and structural changes to meet the needs arising from isolating inpatients and to protect healthcare workers [2]. Isolation is the priority course of action in these cases. However, the negative repercussions of isolation on patients must be taken into account, as they are associated with negative psychological effects, such as risk of developing anxiety and depression; the occurrence of a greater number of adverse events, such as falls; and reduced contact with healthcare workers [3,4]. Isolation inevitably entails limited contact with other patients, disruption of daily life, no visitors, and less interaction with hospital staff. These restrictions may lead to feelings of loneliness, neglect, social exclusion, and stigmatisation [5].

Given the rapid rise in the numbers of infected patients requiring hospitalisation, units admitting patients with suspected or confirmed COVID-19 have had to make a great effort to adapt to emerging needs in terms of material resources, newly hired personnel, and new protocols and procedures required to tackle the pandemic. COVID-19 has led to changes in nurse staffing and increased workload for nurses, which may have negatively affected the quality of nursing records [6]. The work environment is a conditioning factor in the implementation and use of electronic records, which means it may influence their quality and levels of completion [7].

If properly documented in the patient’s clinical record, the following measures are possible: provision of pressure ulcer (PU) care through early identification of patients at risk of developing ulcers; recording of the number of PUs, if any; fall prevention by assessing the risk of falling during hospitalisation; analysis of the causes of a patient’s fall; and identification of socially at-risk patients. Records intended to assess and identify risks among inpatients are essential for developing evidence-based initiatives capable of preventing such risks. Patient assessments using scales and interventions must be conducted regardless of the clinical environment and should be the same for all patients, whether or not they are in isolation. When a patient’s condition is critical or complex, further nursing care is needed [8], and it is essential that this care is recorded.

A unified approach could help frontline healthcare professionals to standardise data collection and increase the efficiency and performance of their work. Documentation is crucial for the communication of healthcare teams and patient outcome measurement and monitoring [9]. This will require continuous training, regular updates, feedback, support from staff, and regular monitoring of records [10].

A number of studies have shown that nurses’ workload is significantly associated with the quality of care provided [11] and that nurses’ workload and patient deaths are positively correlated [12]. The time required to keep electronic records in their daily work can be a source of frustration and exhaustion for nurses, who experience it as additional workload [13]. Nursing records are an important clinical resource, as they are necessary for assessing the delivery of care, enhancing the quality of nursing care, and improving nurses’ work environment and workload [14].

Previous experiments have shown that improving training and implementing best clinical practices is helpful in increasing the completion of nursing records in clinical records and in integrating said completion into nurses’ routine work procedures [15]. 

Further studies and comprehensive tools are needed to analyse this phenomenon in greater depth. There is a need for studies assessing the impact of isolation on safety indicators, in addition to psychological aspects, while taking into account potential collateral damage caused by isolation.

The objective of this study is to describe the impact of the first wave of the COVID-19 pandemic on levels of adherence to the completion of nursing records that document the risk of developing PUs, falling, and risk of social vulnerability among hospitalised patients in isolation.

## 2. Materials and Methods

### 2.1. Study Design

This is an observational pre–post comparison study on the nursing records of patients hospitalised in a tertiary care hospital in the public healthcare system before and during the first phase of the COVID-19 pandemic.

### 2.2. Sampling and Participants

The study population consisted of the records of 1205 inpatients: 568 patients admitted to the Internal Medicine, Vascular Surgery, Otorhinolaryngology, and Cardiology inpatient units at a University Clinical Hospital between 1 and 29 February 2020 (prior to the pandemic) and 637 patients admitted to these same units between 15 March and 15 April 2020 with COVID-19 diagnoses (during the peak of the first wave of the pandemic). These units were repurposed to treat patients with COVID-19 from the second half of March.

### 2.3. Data Collection

Data collection took place in two phases (pre- and post-COVID-19 at the same units), which assessed the completion of electronic records, including the Braden, Downton, and Gijón scales.

Risk of developing PUs was measured using the Braden scale. Scores range from 6 to 26. Scores below 12 points indicate a high risk of developing PUs; scores between 13 and 14 indicate a moderate risk; and scores between 15 and 18 indicate a low risk [16]. The risk of falling was measured using J.H. Downton’s scale. Scores range from 0 to 14, with scores ≥ 3 indicating risk of falling [17]. Socio-familial risk among patients aged 75 or older was measured using the socio-familial Gijón scale. Scores range from 5 to 25. Scores ≤ 8 indicate a low risk; scores from 8 to 9 indicate a moderate risk; and scores ≥ 10 indicate a high risk [18]. 

Phase 1 took place between 1 and 29 February 2020. The electronic records of patients hospitalised before the pandemic were collected. Phase 2 took place between 15 March and 15 April 2020 (during the first wave of the pandemic). The electronic records of patients admitted for COVID-19 were collected.

In both phases, the following variables were also analysed: sex, age, mean length of stay, falls, number of PUs, and places where the PUs originated.

### 2.4. Data Analysis

All statistical analyses were performed using IBM SPSS v. 24.0 software (IBM, Armonk, New York, NY, USA). Quantitative variables were described using means, standard deviations, and minimum and maximum scores. Qualitative variables were described by their distribution of frequencies. Associations between qualitative variables were analysed using Pearson’s chi-squared test. Quantitative values were compared using Student’s *t*-test for paired samples or using ANOVA for more than two samples. The statistical significance threshold for all tests was set at *p* < 0.05.

## 3. Results

The records of 1205 patients were analysed, 54.8% of whom were men and 45.2% women, with a mean age of 70.46 (SD = 16.1) years. In total, 47% of patients were admitted in February 2020 with a mean stay of 9.42 (SD = 2.1) days, and 53% of patients were admitted between 15 March 2020 and 15 April 2020 with a mean stay of 9.18 (SD = 2.8) days. There were no differences between the two periods by sex. By age group, 32.7% were ≤65 and 67.3% > 65 years. However, differences in age were identified. In phase 1, the number of women was 252 and that of men was 316. In phase 2, there were 293 women and 344 men. Regarding the age group, the group ≤ 65 years in phase 1 consisted of 166 patients and 228 patients in phase 2. The group > 65 years in phase 1 consisted of 402 patients and 409 patients in phase 2. The mean age decreased considerably, from 72 (SD = 17) years to 69 (SD = 15) years among patients who had tested positive for COVID-19 (*p* = 0.02).

The degree of completion of the Braden, Downton, and Gijón scales decreased significantly in phase 2 with respect to phase 1 (*p* < 0.001). See Table 1.

The level of adherence to the completion of the records did not show statistically significant differences between the two phases in terms of sex.

The completion of the Braden and Downton scales was significantly higher in the first phase for the group over 65 years (0.012 and *p* = 0.003); see Table 2.

Assessment records using the Downton and Gijón scales revealed scores consistent with a higher risk of falling and social vulnerability in phase 1 compared to phase 2. However, the risk of developing PUs during hospital stay increased in phase 2; see Table 3. 

The number of PUs recorded decreased in phase 2 (phase 1: *n* = 60; 76% vs. phase 2: *n* = 21; 26%; *p* < 0.001). However, an increase in the number of patients who developed PUs during their hospital stay was also observed in phase 2 compared to phase 1 (phase 1: *n* = 7; 33.3% vs. phase 2: *n* = 15; 25%; *p* = 0.033).

Regarding the number of falls, six falls were recorded in each of the study periods, with a total of 12 (phase 1: *n* = 6; 1.05% vs. phase 2: *n* = 6; 0.94%; *p* > 0.005). No statistical significance was observed given the small sample size of patients who experienced a fall during these periods.

Regarding the analyses of demographics (age group and sex), completion level of scale records, and scores, the completion of the Braden and Downton scales was significantly higher in the first phase for the group over 65 years (0.012 and *p* = 0.003). No statistically significant differences were observed when comparing risks between phase 1 and phase 2 in terms of sex or age; see Table 4.

## 4. Discussion

This study reports a decrease in the completion of nursing records assessing the risk of developing PUs, experiencing falls, and social vulnerability among inpatients with COVID-19 in isolation.

The COVID-19 pandemic has resulted in a clear decrease in adherence to the completion of the Braden, Downton, and Gijón scales. Nurses’ failure to record their work does not necessarily mean that they do not complete this work. When nurses have greater workloads, they prioritise their interventions by attaching greater importance to patient care than to recording activities [19]. This may explain why the number of records decreased during the pandemic. This is especially relevant considering that we are comparing the same units and the same staff between two periods, pre- and post-COVID-19. The literature has discussed the unintended effects that can result from the need for strict isolation during the pandemic, which may include incomplete patient documentation [20].

The risk of developing PUs during hospital stays increased in phase 2, as did the number of patients who developed PUs during their hospital stay. Nursing records are a necessary clinical source of relevant information for assessing nursing care, so incomplete or missing records hinder health decision making [14] and, as in this case, hinder health decision making regarding COVID-19 patients. A decrease in the frequency of assessment of the risk of developing PUs using the Braden scale may represent a barrier to early detection and regular risk assessment [21]. 

The levels of risk of falling and risk of social vulnerability of patients admitted to hospital during phase 2 were lower than during phase 1. The occurrence of adverse events in hospitals depends, to a great extent, on the mean length of stay. In this case, the mean lengths of hospital stays were similar in the two study periods, meaning that this factor could not have influenced the analysis. However, the mean age of patients admitted to hospital during the first wave of the COVID-19 pandemic was lower than it was prior to the pandemic. This may be linked to the Downton and Gijón scale scores showing a greater risk of falling and experiencing social vulnerability prior to the pandemic than during the first wave of the pandemic, which may suggest that age, rather than isolation, is the risk factor in this case. The availability of assessment tools that include age as a predictor of risk will help to identify at-risk patients more accurately [22]. The decrease in mean age in phase 2 could be explained by the fact that the samples may have changed markedly as the units were used for unplanned care of COVID-19 patients and may not represent the types of patients in each unit prior to COVID-19. 

Nurses have been deeply involved in the prevention and management of the COVID-19 pandemic in a health system capable of dealing with public health emergencies. Assessment records of the risk of developing PUs must be included in the standardisation of care in new hospital departments and in ongoing improvements to contingency plans [23]. A number of studies associate the risk of developing PUs with severe patient distress in the form of vomiting, shortness of breath, severe pain at rest, urinary problems, and low albumin levels in laboratory tests [21]. This is a clinical scenario similar to that of patients with COVID-19, which justifies the use of the Braden scale in all cases. The decrease in the number of PUs recorded may also be explained or influenced by the decrease in the mean age of the patients admitted to the study units during the pandemic, since age is considered to be a risk factor in developing PUs [21]. However, the number of PUs that occurred while in strict isolation in hospital is particularly worrying, since frequent patient repositioning and mobilisation avoid skin exposure to friction and are vital in PU prevention [24]. The study findings suggest that patient isolation may be associated with a decrease in the frequency of risk assessment and patient mobilisation, leading to decreased PU prevention.

The number of records on the risk of falls among inpatients decreased during the pandemic, which, together with the individual’s risk assessment and personal profile, serves as a risk indicator. The occurrence of falls causing injuries and the risk of falling are related [25]. Fall risk assessments provide information necessary for determining the risk of serious clinical outcomes. Therefore, conducting them enables nurses to identify individuals at high risk of falling and put preventative measures in place [26]. Some studies have found a statistically significant relationship between isolation and the occurrence of falls causing injury [27], which means that risk assessment should be a priority in patient safety. Management of the risk of falling is closely related to recording of this risk, and the correct recording of falls enables the factors contributing to them to be identified [28].

A decrease in the number of records on the risk of social vulnerability was also observed. Hospitals have been overwhelmed and professionals have become exhausted during the pandemic, but it must be borne in mind that unfavourable social and family circumstances can result in prolonged hospital stays, readmissions, reduced quality of life, and lower life expectancy among patients. Therefore, early identification of patients at risk and referral to social services facilitates the integration of patients into the social and family spheres, as health problems in the elderly may result in social and family problems and vice versa [29,30]. 

Analysing the reasons for the decline in the number of nursing records is essential for making care management decisions that prevent these situations from recurring in the event of health system overloads. Quality indicators must be enhanced by improving a number of care-related processes, such as documentation, risk assessments, and reports, which enable nurses to promptly identify at-risk patients and implement specific interventions [31]. Measures that may contribute to preventing a lack of completion of nursing records during the COVID-19 pandemic may include training staff in the use of electronic records systems [15,32] through continued professional development and having nurse managers monitor records to quickly identify any deficiencies [20]. Most importantly, management strategies are needed to allow sufficient time and resources to ensure that nurses are able to effectively perform all important aspects of care, setting staffing levels at the necessary average. Employing low numbers of permanent staff and relying on temporary staff and redeployments jeopardise quality of care and patient safety [33]. It may be necessary to rearrange shift patterns and allocate new nursing staff to reduce nurses’ workload and improve the quality of nursing care [34]. Further studies are needed to identify the impact of these measures on nursing records in terms of quantity and quality and to assess the effects of work overload on the quality of nursing records and care provided [12]. 

The main limitations of this study may be that the sample is limited to a single hospital at a particular moment in time and the unmeasured differences in patient population between phases. Other limitations include the biases inherent to a retrospective study based on past records, although these records are standardised using validated scales to minimise variability.

## 5. Conclusions

Adherence to the completion of nursing records decreased during the first wave of the pandemic. The quality of care is reflected to a great extent in the clinical records that nurses keep for their patients. 

The decrease in the number of nursing records in cases of isolation should prompt the directors of healthcare facilities and healthcare workers to reflect on the need to take measures to encourage recording of risk assessments. 

There is a need for further studies on potential solutions to these situations.

## Figures and Tables

**Table 1:** The degree of completion of the Braden, Downton, and Gijón scales recorded between study phases 1 and 2.

	Study Periods						
	Phase 1		Phase 2		Total		
Number of Patients Assessed	*n* = 568		*n* = 637		*n* = 1205		
Nursing Records Completed	*n*	%	*n*	%	*n*	%	*p*-Value
Braden scale	498	87.7	350	54.9	848	100	<0.001
Downton scale	338	59.5	231	36.3	569	100	<0.001
Gijón scale	224	62.7	133	37.3	357	100	<0.001

**Table 2:** Analyses of completion of scales for age group and sex.

	Phase	Sex				*p*-Value	Age				*p*-Value
		Man		Woman			≤65		>65		
		*n*	%	*n*	%		*n*	%	*n*	%	
Braden scale	1	274	58.9%	224	58.5%	0.897	144	52.6%	354	61.7%	0.012
	2	191	41.1%	159	41.5%		130	47.4%	220	38.3%	
Downton scale	1	184	59.5%	154	59.2%	0.939	73	49.0%	265	63.1%	0.003
	2	125	40.5%	106	40.8%		76	51.0%	155	36.9%	
Gijón scale	1	114	62.3%	110	63.2%	0.857	18	54.5%	206	63.6%	0.306
	2	69	37.7%	64	36.8%		15	45.5%	118	36.4%	

**Table 3:** Mean scores for the Braden, Downton, and Gijón scales between study phases 1 and 2.

	Phase 1 Mean (SD ^†^)	Phase 2 Mean (SD ^†^)	*t*-Test for Independent Samples			*p*-Value
			Differences in Means	95% CI		
				Lower Bound	Upper Bound	
Braden scale	17.83 (4.47)	18.45 (4.41)	−0.62	−1.23	−0.01	0.04
Downton scale	2.78 (1.67)	1.87 (1.50)	0.91	0.65	1.17	<0.001
Gijón scale	6.88 (2.44)	3.68 (1.79)	3.20	2.76	3.64	<0.001

^†^ Standard deviation.

**Table 4:** Analyses of risk scale scores for age group and sex.

	Phase	Sex	Mean (SD)	*p*-Value	Age	Mean (SD)	*p*-Value
Braden scale	1	Man	18.21 (4.35)	0.278	≤65	20.47 (3.21)	0.960
		Woman	17.37 (4.57)		>65	16.76 (4.46)	
	2	Man	18.53 (4.09)	0.221	≤65	20.76 (3.28)	
		Woman	18.36 (4.78)		>65	17.09 (4.43)	
Downton scale	1	Man	2.57 (1.6)	0.221	≤65	1.71 (1.29)	0.825
		Woman	3.05 (1.73)		>65	3.08 (1.65)	
	2	Man	1.81 (1.52)	0.221	≤65	1 (0.99)	
		Woman	1.95 (1.48)		>65	2.3 (1.52)	
Gijón scale	1	Man	6.82 (2.68)	0.470	≤65	6.5 (3.19)	0.947
		Woman	6.94 (2.18)		>65	6.91 (2.37)	
	2	Man	3.45 (1.49)	0.470	≤65	3.27 (0.59)	
		Woman	3.92 (2.05)		>65	3.73 (1.88)	

## Data Availability

The data presented in this study are available on request from the corresponding author.
